# Unstructured protein domains stabilize RNA binding and mediate RNA folding by AUF1

**DOI:** 10.1016/j.jbc.2025.108442

**Published:** 2025-03-25

**Authors:** Nina C. Lee, Haley H. Tilley, Grace A. Acle, Patrick J. McGinnis, Gerald M. Wilson

**Affiliations:** Department of Biochemistry and Molecular Biology, Center for Biomolecular Therapeutics, and Marlene and Stewart Greenebaum Comprehensive Cancer Center, University of Maryland School of Medicine, Baltimore, Maryland, USA

**Keywords:** AU-rich element, AU-rich element RNA-binding protein 1 (AUF1), fluorescence anisotropy, fluorescence resonance energy transfer (FRET), RNA binding protein, RNA folding, thermodynamics

## Abstract

AUF1 is an RNA-binding protein that targets AU-rich elements, *cis*-acting regulatory sequences commonly enriched in mRNAs encoding inflammatory mediators and oncoproteins. AUF1 post-transcriptionally regulates gene expression by modulating the stability and/or translational efficiency of mRNA targets in a context-specific manner; however, the mechanisms by which AUF1 directly engages RNA substrates and mediates regulatory outcomes remain largely unknown. The purpose of this study was to define the biochemical basis for RNA recognition by AUF1 using the smallest protein isoform (p37^AUF1^) as a model. AUF1 contains two tandem RNA recognition motifs (RRMs), common RNA-binding domains that stabilize the formation of many ribonucleoprotein complexes. Using quantitative fluorescence anisotropy–based assays, we observed that p37^AUF1^’s tandem RRM domain only weakly binds AU-rich element substrates. Testing a panel of protein mutants revealed that the N- and C-terminal flanking domains each make modest but similar contributions to stabilization of both the initial RNA:protein complex and a subsequent protein-binding event. However, focused protein truncations showed that residues immediately N-terminal of the RRMs were vital for high affinity binding, but only in the context of the C-terminal domain. The C-terminal domain was also required for protein-induced RNA remodeling; both this function and its ribonucleoprotein-stabilizing role involve nonbase-specific contacts with RNA upstream of the AU-rich motif. Finally, our data suggest that the C-terminal domain is intrinsically disordered but may undergo a conformational change upon interaction with RNA ligands. Together, these findings reveal distinct roles for flanking protein domains in RNA binding and remodeling by AUF1.

Tight control of gene expression is vital for cellular homeostasis and function and can be regulated at many different points including transcription, RNA turnover, translation, and at posttranslational levels. A key metric controlling protein abundance is the steady-state level of its encoding mRNA, which is balanced by the rates of mRNA synthesis and degradation (1, 2, 3). However, steady-state mRNA levels are also altered in response to a wide variety of cellular stimuli. At post-transcriptional levels of gene regulation, these changes are frequently coordinated among mRNA subpopulations through conserved *cis*-acting sequence elements within their 3′ UTRs (3, 4, 5). These *cis*-acting sequences are targeted by *trans*-acting factors, which can include RNA-binding proteins, long noncoding RNAs, and miRNAs. One well-studied family of *cis-*acting sequences are the AU-rich elements (AREs), which often contain one or more AUUUA motifs within a U-rich background and regulate mRNA stability and/or translational efficiency (4, 6, 7). AREs are relatively common with variants identified in as many as 22% of mRNA 3′UTRs in humans and are particularly enriched in transcripts that encode pro-inflammatory and pro-oncogenic factors (8).

The subset of RNA-binding proteins that target AREs are denoted as ARE-binding proteins (AUBPs) (4, 9). These proteins can positively or negatively modulate the stability and/or translation of ARE-containing mRNAs and can be regulated by a variety of cellular signals. However, dysregulation of AUBP expression or function can also lead to widespread pro-inflammatory or protumorigenic effects, due to the resulting consequences on the production of oncogenes, chemokines, and cytokines encoded by ARE-containing mRNAs (10, 11). Accordingly, abnormal accumulation of ARE-containing mRNAs and their encoded protein products are often found in cancers and chronic inflammatory conditions, indicating their potential utility as biomarkers or therapeutic targets (4, 10).

One important AUBP is ARE/poly(U)-binding/degradation factor 1 (AUF1), also known as heterogeneous nuclear ribonucleoprotein D. It is expressed as four isoforms, distinguished by their apparent molecular weights, and are known as p45^AUF1^, p42^AUF1^, p40^AUF1^, and p37^AUF1^ (12, 13, 14). These isoforms are formed *via* alternative pre-mRNA splicing that removes exon 2 in the cases of p37^AUF1^ and p42^AUF1^ or exon 7 for p37^AUF1^ and p40^AUF1^. All isoforms contain two tandem, centrally positioned RNA recognition motifs (RRMs) that are flanked by N- and C-terminal domains. Each isoform has intrinsic differences in subcellular localization, RNA-binding activity, and oligomerization potential (15, 16). All AUF1 isoforms can form oligomers on AREs using a sequential protein-binding model, where the limit on oligomer size depends on the length of the ARE substrate (16, 17, 18). In cells, AUF1 binding can trigger several different fates for mRNA targets including destabilization, stabilization, or enhancement of translation (14, 19, 20). However, AUF1’s targets are not restricted to mRNAs; it can also bind and regulate the levels of long noncoding RNAs (21, 22, 23), as well a subset of miRNAs, where it may enhance loading into the RISC complex (24). Despite the evidence that AUF1 has important roles in the control of inflammation and cancer (10, 25, 26, 27), the biochemical mechanisms by which it binds and subsequently regulates RNA substrates are largely unknown.

A previous study delineated roles for select AUF1 domains on its RNA-binding activity (28) but was limited by the technology and other information available at the time. For example, binding was quantified using electrophoretic mobility shift assays (EMSAs), but comparisons with later studies using fluorescence-based approaches at equilibrium revealed that EMSAs underestimate AUF1 binding to many RNA ligands, likely due to rapid dissociation of these complexes following gel loading owing to their very rapid off-rates (17). Also, the limits of AUF1’s RRM domains were not structurally defined, which complicated the assignment of RRM contributions to RNA binding. Even now, structural studies of AUF1 remain very limited. NMR-derived structures of individual RRMs and a crystal structure of the N-terminal RRM alone (29, 30, 31) have helped to assign the limits of each RRM based on the adoption of canonical βαββαβ folds but yielded few details to clarify how they engage and discriminate RNA ligands. Furthermore, no structural information is available regarding the N- and C-terminal domains of AUF1.

Given the dearth of information regarding AUF1 structure, this study focused on the biochemical roles of specific AUF1 domains on its RNA-binding and RNA-remodeling activities. We used the smallest AUF1 isoform (p37^AUF1^) as a model, as it has the strongest RNA-binding affinity and most potent mRNA-destabilizing ability in cell models (16, 32). It was also aided by a prior biochemical dissection of the RNA requirements for high affinity p37^AUF1^ binding, which allowed discrimination of the initial AUF1 recruitment step from subsequent protein oligomerization steps on RNA substrates (18). Here, we used a variety of quantitative fluorescence-based techniques to define the contributions of the RRMs and flanking domains to the RNP assembly functions of AUF1, including RNA-binding affinity, RNA-induced protein oligomerization, and local RNA conformational remodeling. Our data indicate that select regions within intrinsically disordered flanking protein domains make major contributions to stabilizing AUF1 RNP complexes. Furthermore, the p37^AUF1^ C-terminal domain is entirely responsible for modulating the structure of bound RNA ligands, demonstrating key roles for sequences beyond the RRMs in execution of AUF1 function.

## Results

### The N- and C-terminal domains of p37^AUF1^ both stabilize initial RNP complex formation

Historically, recombinant AUF1 proteins have been efficiently purified *via* immobilized metal affinity chromatography using an N-terminal His_6_ tag (16, 28, 33, 34). However, to our knowledge, no studies have yet confirmed that the His_6_-tag does not alter the RNA-binding activity of full-length p37^AUF1^. To test this, the His_6_-tag was excised from purified His_6_-p37^AUF1^ using enterokinase (EK) (Fig. S1*A*). ARE-binding activity was then measured by fluorescence anisotropy using a fluorescein (Fl)-tagged RNA substrate optimized for high affinity AUF1 binding called ARE1-Fl (Table 1), consisting of a 15-nt core AU-rich sequence but flanked by a 3′ G and a 19-nt 5′-extension previously shown to stabilize p37^AUF1^ RNP complexes in a largely sequence-independent manner (18). Affinity of the EK-cleaved p37^AUF1^ for the ARE1-Fl RNA ligand (*K*_*d-app*_ = 15 ± 2 nM, Fig. S1*B*) did not significantly differ from that of the His_6_-tagged version (*K*_*d-app*_ = 19 ± 3 nM), minimizing concerns that the His_6_-tag would substantially alter the RNA-binding activities of AUF1 proteins tested in this study.

**Table 1:** RNA substrates used in this study

Name	Sequence (5′→3′)^a^
ARE1^b^	CCCCUGGCUCACAAAUACCUUAUUUAUUUAUUUAG
ARE2	GUGAUUAUUUAUUAUUUAUUUAUUAUUUAUUUAUUUAG
ARE_15_-G	UUAUUUAUUUAUUUAG
Rβ	UGGCCAAUGCCCUGGCUCACAAAUACCACUG

a RNA substrate variants containing 5′-linked cyanine-3 (Cy3) or fluorescein (Fl) groups are indicated by relevant prefixes, where applicable. Similarly, RNA substrates containing 3′- fluorescein groups are suffixed where appropriate by “Fl.” RNA substrates containing 5′-biotin tags are prefixed with “B.”

b For the ARE1 and ARE_15_-G substrates, the core ARE sequences are underlined.

**Figure 1:**
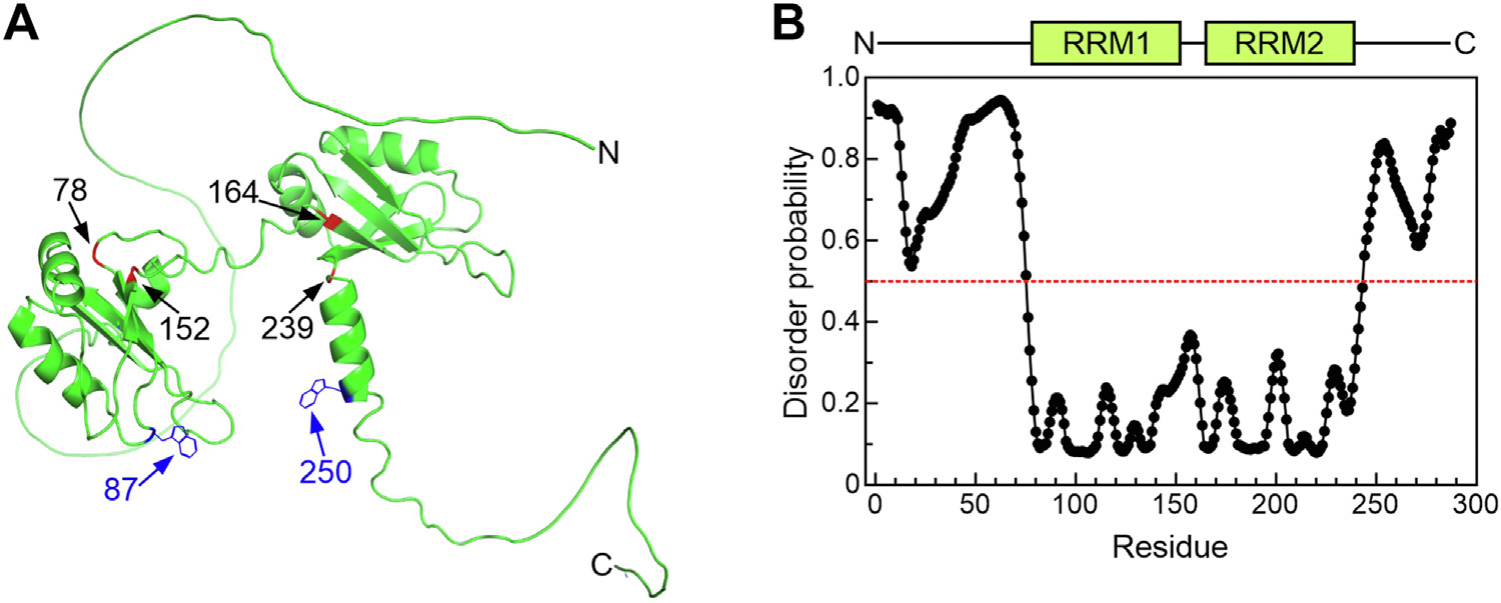
**Computational predictions of p37^AUF1^ structure.***A*, AlphaFold2 prediction of p37^AUF1^ structure. The limits of each RRM are marked in *red* and labeled in *black*. The locations of the two tryptophan residues of p37^AUF1^ are in *blue*. *B*, PrDOS intrinsic disorder prediction graph for p37^AUF1^. The *red dotted* line denotes the disorder cutoff (0.5). Residues grouped above the line are predicted to be disordered, while ones below the line are ordered. Corresponding locations of the flanking domains and RRMs are indicated in the schematic (*top*).

Given the absence of any reported structural information on AUF1 domains beyond the RRMs, the sequence of p37^AUF1^ was input into AlphaFold2 (35, 36) and PrDOS, an independent software package that predicts intrinsically disordered regions (37). Both programs predicted two robustly ordered RRM modules, but no defined structures for either flanking domain, consistent with the hypothesis that they are intrinsically disordered (Fig. 1). The boundaries of the RRM modules predicted by AlphaFold2 (numbered in black in Fig. 1*A*) were consistent with those resolved by NMR (29, 30) and are used hereafter in this study to define the limits of these motifs.

**Figure 2:**
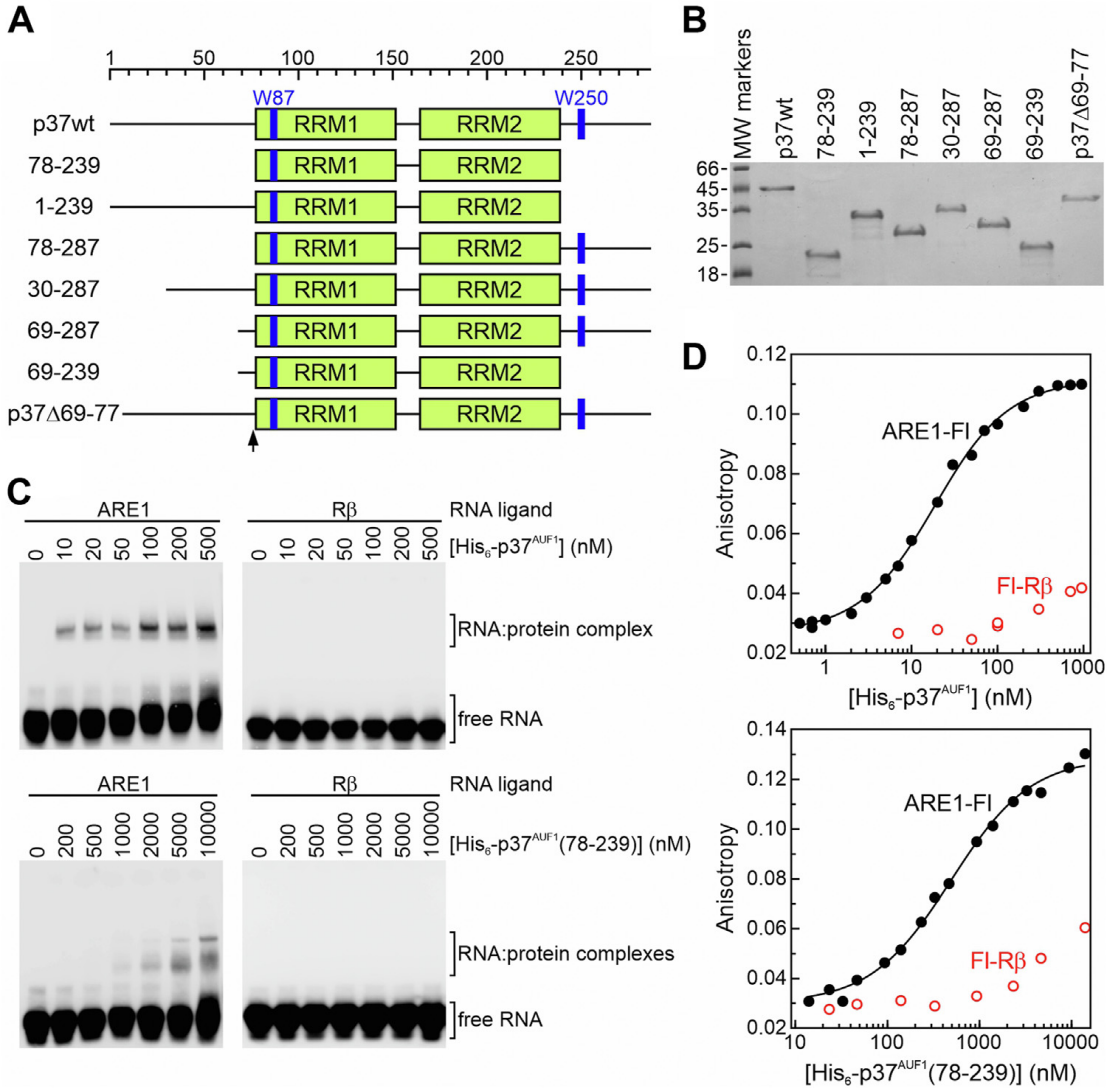
**RNA-binding activities of His_6_-p37^AUF1^ and select mutants.***A*, schematics of WT p37^AUF1^ and domain mutant proteins used in this study. The flanking domains and RRMs (*green boxes*) are to scale. Tryptophan residues are marked in *blue* at positions 87 and 250, and an internal deletion indicated *via* a *black* arrow. *B*, Coomassie Blue–stained SDS-PAGE gel of purified His_6_-p37^AUF1^ and mutant proteins. Molecular weight markers (in kDa) are shown on the *left*. *C*, EMSAs of His_6_-p37^AUF1^ (*top*) and His_6_-p37^AUF1^(78-239) (*bottom*) binding to either B-ARE1 (*left*) or B-Rβ (*right*) ligands. *D*, fluorescence anisotropy–based assays of His_6_-p37^AUF1^ (*top*) and His_6_-p37^AUF1^(78-239) (*bottom*) binding ARE1-Fl (*black, closed circles*) or Fl-Rβ (*red, open circles*) RNA ligands. Isotherms with ARE1-Fl were solved using the single-site binding model (Equation 1) described under Experimental procedures. Apparent dissociation constants are listed in Table 2.

To determine the contribution of the tandem RRM domain to RNA binding, His_6_-p37^AUF1^(78-239) was constructed and its affinity for the ARE1 substrate measured and compared to the WT protein (Fig. 2, *A* and *B*). Initially, EMSAs following UV-crosslinking were used to qualitatively evaluate binding activity. His_6_-p37^AUF1^ largely formed a single shifted species with biotin (B)-tagged ARE1 (Fig. 2*C*, *top left*), consistent with a previous report and suggesting a single principal-binding event (18). Quantitative analyses of protein binding to the fluorescent ARE1-Fl ligand using fluorescence anisotropy also supported a single RNP complex for His_6_-p37^AUF1^ based on high coefficients of determination (*r*^2^ > 0.99) and random distributions of residuals (*p* > 0.05) when data were fit to a single-site binding model (Fig. 2*D*, *top*). In contrast, His_6_-p37^AUF1^(78-239) required much higher protein concentrations to generate RNP complexes detectable by EMSA (Fig. 2*C*, *bottom left*), suggesting a significant penalty in binding affinity. Additionally, His_6_-p37^AUF1^(78-239):ARE1 RNPs appeared to migrate as two closely migrating bands formed at similar protein concentrations. Given the significantly smaller size of His_6_-p37^AUF1^(78-239) versus the full-length protein, these bands could conceivably represent two protein-binding events on a single RNA ligand or individual binding events at different locations on the substrate. However, anisotropy-based analyses of His_6_-p37^AUF1^(78-239) binding to the ARE1-Fl substrate were well resolved by a single-site binding model (Fig. 2*D*, *bottom*; *r*^2^ > 0.99 and random distributions of residuals (*p* > 0.05)). Analyzing these data using a cooperative binding model (Equation 3) revealed a Hill coefficient that was not significantly different than 1 (*data not shown*), indicating no detectable allostery between protein binding events. Together, these observations suggest that, even if His_6_-p37^AUF1^(78-239) does form two distinct complexes on the ARE1-Fl ligand, they are likely independent and thermodynamically equivalent and thus both quantifiable by a single *K*_*d-app*_. Neither His_6_-p37^AUF1^(78-239) nor the WT protein formed a detectable complex with a non-ARE substrate (B-Rβ) in EMSA experiments indicating that sequence specificity for ARE ligands was retained in the tandem RRM domain (Fig. 2*C*, *right panels*).

Quantitative comparisons of RNA-binding affinity were performed by calculating apparent dissociation constants (*K*_*d-app*_) from fluorescence anisotropy isotherms. WT His_6_-p37^AUF1^ bound the ARE1-Fl ligand with *K*_*d-app*_ = 19 ± 3 nM, but binding by the tandem RRM domain alone was 25-fold weaker (*p* < 0.0001, Table 2), indicating major roles for one or both flanking protein domains in stabilizing complexes with this RNA substrate. This differed from observations made for several other RRM-containing proteins, where the RRM domain(s) alone were largely sufficient to form high-affinity RNP complexes (38, 39, 40). Neither His_6_-p37^AUF1^ nor the 78-239 mutant protein showed appreciable binding to the fluorescent Fl-Rβ control RNA (Fig. 1*D*), further indicating that weak ARE binding by the tandem RRM domain was nonetheless sequence-specific.

**Table 2:** Affinities of p37^AUF1^ and indicated mutants for the ARE1-Fl RNA substrate

Protein^a^	*K*_*d-app*_^b^ (nM)	*n*	Δ*G*°^c^ (kcal/mol)	ΔΔ*G* vs wt (kcal/mol)
p37wt	19 ± 3	7	−10.5	0
78–239	505 ± 34	6	−8.6	1.9
1–239	101 ± 8	5	−9.5	1.0
78–287	214 ± 18	5	−9.1	1.4
30–287	27 ± 1.4	3	−10.3	0.2
69–287	59 ± 9	4	−9.9	0.6
69–239	527 ± 54	4	−8.6	2.0
p37 (Δ69–77)	89 ± 11	3	−9.6	0.9

a Proteins described in Figure 2*A*. All include N-terminal His_6_ tags.

b Apparent equilibrium dissociation constants were resolved using fluorescence anisotropy–based binding assays by nonlinear regression to Equation 1 where *K*_*d-app*_ = 1/*K* as described in the legend for Figure 2*D* and are listed as the means ± SD of *n* independent experiments.

c Free energy of protein binding to the ARE1-Fl ligand was calculated as Δ*G*° = -*RT*ln*K*.

**Figure 3:**
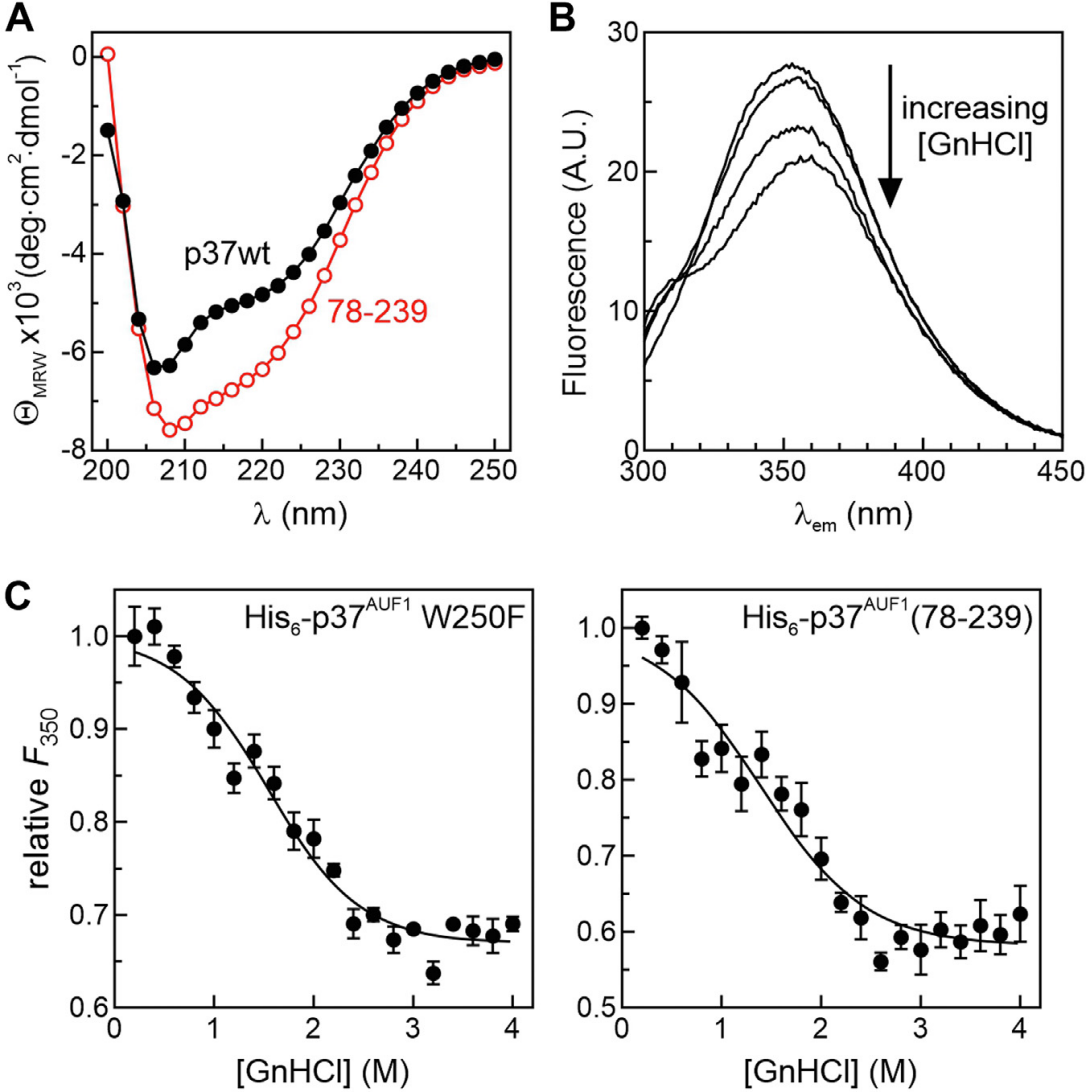
**Flanking sequence effects on folding of the tandem RRM domain.***A*, CD spectra of His_6_-p37^AUF1^ (*black, closed circles*) and His_6_-p37^AUF1^(78-239) (*red, open circles*). *B*, fluorescence emission spectra of His_6_-p37^AUF1^ W250F (8 μM, λ_ex_ = 280 nm) after incubation in 0.2, 1, 2, or 3 M GnHCl. *C*, representative plots showing relative changes in fluorescence emission at 350 nm from His_6_-p37^AUF1^ W250F (*left*) and His_6_-p37^AUF1^(78-239) (*right*) as a function of GnHCl concentration. Points are the mean ± SD of five technical replicates per sample. Plots were analyzed to solve for thermodynamic parameters describing the folded stability of each protein by nonlinear regression using Equations 5 and 6 as described in Experimental procedures. Data compiled from three independent replicate experiments are listed in Table 3.

The substantial energetic penalty in ARE binding by His_6_-p37^AUF1^(78-239) presents two potential explanations. First, sequences flanking the tandem RRM domain may play direct roles in binding RNA and stabilizing RNP complexes. Alternatively, the poor RNA-binding affinity of the tandem RRM domain could be due to protein misfolding in the absence of flanking sequences. To test the misfolding hypothesis, two orthogonal methods were used to analyze folding of the His_6_-p37^AUF1^(78-239) protein. First, CD measurements were taken to evaluate the overall secondary structure composition of both His_6_-p37^AUF1^ and His_6_-p37^AUF1^(78-239) (Fig. 3*A*). Both proteins had similar CD spectra consistent with the characteristic profile for RRMs, where there are two minima: one at 208 nm and a shallower one at 222 nm. These features indicate the presence of separate β-strands and α-helices (41), constituent secondary structures within properly folded RRMs (42), and suggest that the global structure of the RRMs is similar in both the full-length protein and His_6_-p37^AUF1^(78-239). Differences in amplitude between the CD spectra are likely caused by the flexible flanking domains, since disordered sequences contribute very little ellipticity in the 210 to 250 nm range (43, 44). The second approach was a fluorescence-based protein denaturation experiment. His_6_-p37^AUF1^ has only two tryptophan residues, one at position 87 (W87) within the N-terminal RRM and the other at position 250 (W250) in the C-terminal domain (Fig. 2*A*). Since W87 is shared between both full-length His_6_-p37^AUF1^ and His_6_-p37^AUF1^(78-239), this residue was selected to evaluate local changes in the RRM structure. To remove complications from W250 fluorescence in the WT protein, a mutant substituting phenylalanine at this position (His_6_-p37^AUF1^ W250F) was generated and purified (Fig. S2*A*). Fluorescence anisotropy–based binding assays demonstrated that this mutation had no effect on protein affinity for the ARE1-Fl RNA substrate (*K*_*d-app*_ = 22 ± 2 nM, Fig. S2*B*). Protein denaturation analyses were then performed using titrations of guanidine hydrochloride (GnHCl) and monitored by decreases in W87 fluorescence emission (λ_max_ = 350 nm) observed during protein unfolding (Fig. 3*B*). Plots were analyzed using the linear extrapolation model described under “Experimental procedures” (Fig. 3*C*) to resolve the free energy of denaturation in the absence of denaturant (Δ*G*_*uw*_) and the sensitivity of the free energy of denaturation to GnHCl (*m*_*eq*_). Comparing results from experiments with His_6_-p37^AUF1^ W250F and His_6_-p37^AUF1^(78-239) revealed no significant differences in either parameter (Table 3), indicating that protein domains near W87 are folded with similar energetics. Together with the CD data, these results give strong evidence against the misfolding hypothesis. Therefore, the energetic contributions of AUF1 flanking domains to RNP stability are likely the result of direct contacts with the RNA ligand.

**Table 3:** Thermodynamic parameters describing GnHCl-induced unfolding of p37^AUF1^ W250F and p37^AUF1^(78-239)

Protein^a^	Δ*G*_*uw*_^b^ (kcal·mol^−1^)	*m*_*eq*_^b^ (kcal·mol^−1^·M^−1^)	*n*
p37 W250F	1.74 ± 0.20	1.18 ± 0.09	3
78-239	1.44 ± 0.17	0.95 ± 0.20	3

a Proteins described in Figure 2*A* and S2*A*. All include N-terminal His_6_ tags.

b The free energy of protein unfolding in the absence of denaturant (Δ*G*_*uw*_) and the sensitivity of the free energy of denaturation to GnHCl (*m*_*eq*_) were resolved from *F*_350_*versus* [GnHCl] plots (Fig. 3) using Equations 5 and 6 and are listed as the means ± SD of three independent experiments.

Based on this model, the contributions of individual flanking domains to AUF1:ARE complex assembly were measured using fluorescence anisotropy–based binding assays. Affinity of recombinant proteins lacking either the C-terminal (His_6_-p37^AUF1^(1-239)) or N-terminal domains (His_6_-p37^AUF1^(78-287), Fig 2, *A* and *B*) for the ARE1-Fl substrate was much weaker than observed with the full-length protein (ΔΔ*G* = −1.0 and −1.4 kcal/mol, respectively, *p* < 0.0001 vs. His_6_-p37wt, Table 2), with removal of the N-terminal domain exerting the slightly larger penalty. Parallel experiments with the Fl-Rβ control ligand showed that deletion of individual flanking domains had no effect on protein selectivity for ARE substrates (*data not shown*).

### Flanking domains of His_6_-p37^AUF1^ make distinct energetic contributions to RNA-induced protein oligomerization

**Figure 4:**
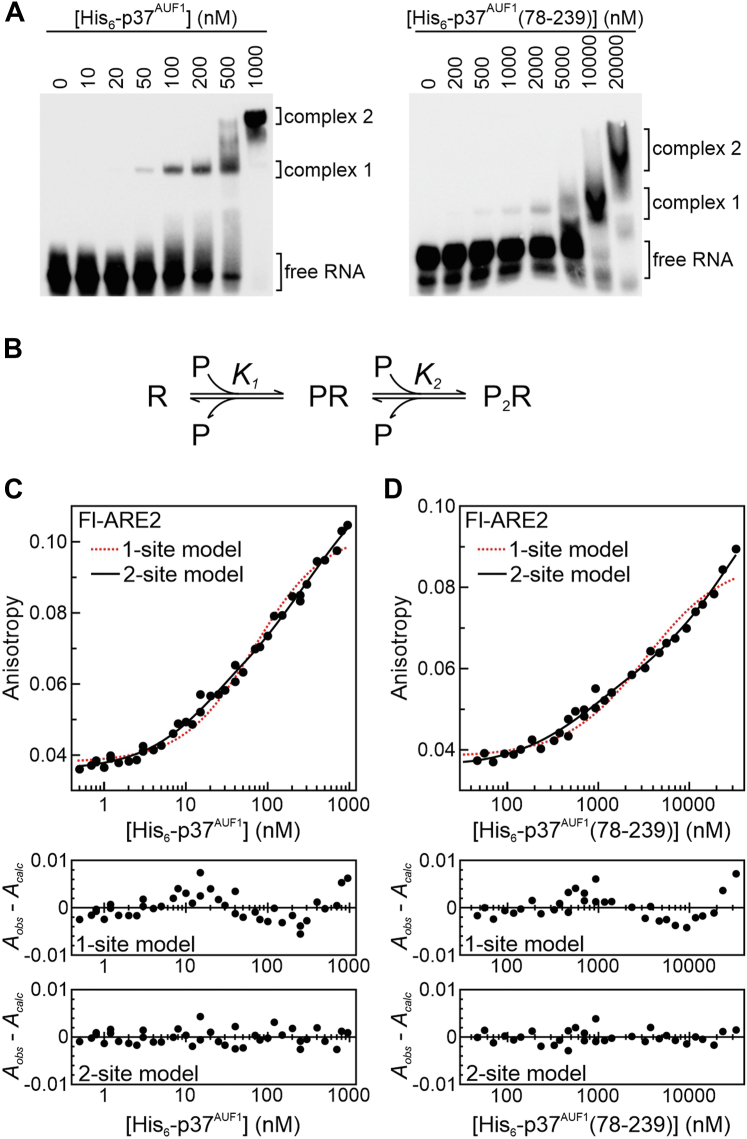
**Contributions of p37^AUF1^ flanking domains to RNA-induced protein oligomerization.***A*, EMSAs of His_6_-p37^AUF1^ (*left*) and His_6_-p37^AUF1^(78-239) (*right*) binding B-ARE2 RNA. Two major complexes were visible in each experiment and are labeled based on order of appearance. *B*, schematic for 2-step protein (P) binding to an RNA substrate (R), defining association binding constants (*K*_*x*_) for each stage of RNP assembly. *C* and *D*, fluorescence anisotropy–based assays of His_6_-p37^AUF1^ (*C*) and His_6_-p37^AUF1^(78-239) (*D*) binding the Fl-ARE2 RNA ligand (*top*) resolved using the single-site binding model of Equation 1 (*red dotted lines*) and two-site binding model of Equation 2 (*black solid lines*). Fitting His_6_-p37^AUF1^(78-239) isotherms to the two-site binding model required fixing the value of the intrinsic anisotropy of the saturated complex (*A*_*P2R*_) in Equation 2. This parameter was set to 0.115 based on the value resolved from two-site binding solutions of full-length p37^AUF1^ binding to this substrate. Residual plots for all regression solutions are in the panels below. Apparent dissociation constants calculated from two-site binding models are listed in Table 4.

Previous studies demonstrated that all AUF1 isoforms form oligomeric structures on extended RNA ligands (16, 17, 28). To determine whether the unstructured p37^AUF1^ flanking domains contribute to protein oligomerization on a longer ARE substrate, RNA-binding activities of His_6_-p37^AUF1^ and His_6_-p37^AUF1^ (78-239) were measured using the ARE2 ligand (Table 1), which includes the ARE from tumor necrosis factor α mRNA. EMSA experiments revealed that both proteins formed two distinct complexes with this RNA substrate (Fig. 4*A*), with the initial complex disappearing as the second forms consistent with the precursor-product relationship expected for oligomerization on a single RNA (Fig. 4*B*) (17). Similar to the ARE1-binding experiments, significantly higher concentrations of His_6_-p37^AUF1^(78-239) were required to form RNP complexes than for His_6_-p37^AUF1^, suggesting that removal of the protein flanking domains severely inhibits RNP assembly at both binding steps. This observation was reinforced by quantitative data from fluorescence anisotropy experiments. Resolution of anisotropy data for His_6_-p37^AUF1^ or His_6_-p37^AUF1^(78-239) binding to the fluorescent Fl-ARE2 ligand using a single-site model yielded nonrandom distributions of residuals (*p* < 0.05), while two-site solutions did not (Fig. 4, *C* and *D*). Furthermore, the sum-of-squares deviations were significantly increased in fits to single-site *versus* two-site binding models for both proteins when compared using the *F* test (*p* < 0.0001). Together, these data indicate that full-length p37^AUF1^ and its isolated tandem RRM domain both form oligomeric complexes on the ARE2 ligand.

Comparing apparent dissociation constants resolved from two-site binding models revealed that deletion of the p37^AUF1^ flanking domains significantly inhibited both the first and second binding steps of RNP assembly on the Fl-ARE2 ligand (Table 4, *cf.* p37wt *vs*. 78-239, *p* < 0.0001). Inclusion of either the N- or C-terminal flanking domains partially restored affinity at the initial binding step (*K*_*d1-app*_) for this substrate, analogous to observations with the single-site ARE1 ligand (Table 2). However, affinity at the second protein-binding step (*K*_*d2-app*_) was almost completely recovered by inclusion of the C-terminal domain (Table 4, *cf.* p37wt *vs*. 78-287), while the N-terminal domain made more modest contributions to stability of the P_2_R complex.

**Table 4:** Multistep binding affinities of p37^AUF1^ and indicated mutants to the Fl-ARE2 RNA substrate

Protein^a^	*K*_*d1-app*_^b^ (nM)	*K*_*d2-app*_^b^ (nM)	*n*	Δ*G°*_*1*_^c^ (kcal/mol)	Δ*G°*_*2*_^c^ (kcal/mol)	ΔΔ*G°*_*1*_ vs wt (kcal/mol)	ΔΔ*G°*_*2*_ vs wt (kcal/mol)
p37wt	16 ± 3	443 ± 83	6	−10.6	−8.7	0	0
78–239	853 ± 48	>10,000^d^	3	−8.3	−5.9	2.3	>2.8^d^
1–239	156 ± 7	2011 ± 446	3	−9.3	−7.8	1.3	0.9
78–287	104 ± 8	663 ± 35	3	−9.5	−8.4	1.1	0.3

a Proteins described in Figure 2*A*. All include N-terminal His_6_ tags.

b Apparent equilibrium dissociation constants were resolved using fluorescence anisotropy–based binding assays by nonlinear regression to Equation 2 where *K*_*dx-app*_ = 1/*K*_*x*_ as described in the legend for Figure 4*C* and are listed as the means ± SD of *n* independent experiments.

c Free energy of protein binding to the Fl-ARE2 ligand was calculated as Δ*G*° = -*RT*ln*K*.

d Because solution of His_6_-p37^AUF1^(78-239) binding to Equation 2 required estimation of the upper asymptote value (*A*_P2R_), the lower limit value for *K*_*d2-app*_ and its associated ΔΔ*G°*_*2*_ are listed.

### A sequence immediately upstream of the tandem RRM domain makes key contributions to RNP stability

The N-terminal flanking domain contributed 1.4 kcal/mol to formation of the PR complex on the ARE1 ligand (Table 2, *cf.* p37wt *vs*. 78-287) and only slightly less on the ARE2 substrate (Table 4). To determine whether RNP stabilization was mediated by specific subsections of this unstructured sequence, additional N-terminal deletions were constructed (Fig. 2, *A* and *B*) and tested for binding to ARE1-Fl RNA. Removing the first 29 residues had minimal impact on RNA-binding affinity (Table 2, *cf.* p37wt *vs*. 30-287), while extending the N-terminal deletion to 68 residues had a modestly larger inhibitory effect (Table 2, *cf.* p37wt *vs*. 69-287, *p* < 0.0001). However, comparing the affinity of His_6_-p37^AUF1^(69-287) *versus* His_6_-p37^AUF1^(78-287) for the ARE1-Fl ligand indicates that inclusion of the nine residues (69-77) immediately upstream of the tandem RRM domain contributes 0.8 kcal/mol to the RNP complex (*p* < 0.0001), over half of the benefit conferred by the complete N-terminal sequence. To corroborate this finding, we constructed a mutant protein lacking only these nine residues (His_6_-p37^AUF1^(Δ69-77)) and measured its ARE-binding affinity. Deletion of this sequence alone inhibited binding to the ARE1-Fl ligand with ΔΔ*G*° = 0.9 kcal/mol relative to the full-length protein (Table 2, *p* < 0.0001), similar to the stabilizing effect described above for these nine residues. These data suggest that amino acids 69 to 77 make specific contributions to binding energy, since juxtaposition of other disordered residues upstream of the tandem RRM domain (*i.e.*, in His_6_-p37^AUF1^(Δ69-77)) does not restore the RNP-stabilizing effect of this sequence.

While the preceding experiments show that amino acid residues 69-78 contribute 0.8 to 0.9 kcal/mol to His_6_-p37^AUF1^ binding to the ARE1-Fl ligand, we next asked whether this sequence was sufficient to enhance protein binding in the absence of the C-terminal domain, which also stabilizes the RNP complex by 1 kcal/mol. This hypothesis was tested by adding this sequence to the tandem RRM domain alone to generate His_6_-p37^AUF1^(69-239) (Fig. 2, *A* and *B*). However, fluorescence anisotropy–based binding assays revealed that residues 69-77 conferred no improvement in affinity for the ARE1-Fl substrate in this context (Table 2, *cf.* His_6_-p37^AUF1^(69-239) *vs*. His_6_-p37^AUF1^(78-239)). Cumulatively, these data indicate that high affinity RNA binding by p37^AUF1^ requires residues from both the N- and C-terminal domains. While the 69-77 sequence makes a particularly strong contribution to complex stability, this enhancement is only observed in the presence of the protein C-terminal domain.

### RNP stabilization *via* nonspecific nucleotides 5′ of the core ARE requires the C-terminal domain of p37^AUF1^

Previous studies defining the RNA determinants for high affinity AUF1 binding revealed two unusual features. First, the initial p37^AUF1^ RNP complex requires a relatively large RNA substrate, as binding significantly weakens on ARE ligands shorter than 30 nucleotides in length (16). This contrasts with other well-known ARE-binding proteins HuR and tristetraprolin, which require only 13 and 9 nucleotides, respectively, to bind with low nanomolar affinities (39, 45). Second, AU-rich sequence is only required for the 3′-half of an RNA ligand for high affinity p37^AUF1^ binding; nucleotides 5′ of this stabilize RNP complexes largely independent of sequence, which led to identification of the ARE1 ligand used in this study (18). Why optimal p37^AUF1^ binding requires such a large and varied stretch of RNA is still unknown. However, our data showing distinct roles for p37^AUF1^ flanking domains in stabilizing RNP complexes prompted the hypothesis that different domains of p37^AUF1^ engage distinct regions of the RNA substrate. Specifically, one or both intrinsically disordered domains may make multiple weak, base-independent interactions with nucleotides 5′ of the core ARE sequence to enhance binding affinity.

To test this hypothesis, we compared the affinity of various His_6_-p37^AUF1^ domain mutants for the ARE1-Fl RNA ligand, which contains the 19-nucleotide 5′ extension, *versus* the ARE_15_-G-Fl substrate, which includes an identical ARE domain and 3′ G residue, but lacking the 5′ extension (Table 1). Removing the 5′ non-ARE sequence weakened binding by full-length His_6_-p37^AUF1^ by 1 kcal/mol (Table 5, *p* < 0.0001), similar to our previous report (18). By contrast, this sequence had a more modest effect on binding by the tandem RRM domain alone (ΔΔ*G*° = 0.3 kcal/mol), suggesting that one or both flanking domains of the protein were required for RNP stabilization *via* the 5′ extended sequence. Similar experiments with other protein mutants revealed that removal of the C-terminal domain largely abrogated the energetic benefit of the longer RNA substrate (Table 5, *cf.* p37wt *vs*. 1-239, 78-287 *vs.* 78-239, and 69-287 *vs.* 69-239). Together, these data indicate that the C-terminal domain of p37^AUF1^ is required for enhancing protein binding to RNA ligands containing 5′ extended sequences.

**Table 5:** Energetic contributions of a 5′-non-ARE sequence to RNP assembly by p37^AUF1^ and indicated mutants

Protein^a^	ARE1-Fl *K*_*d-app*_^b^ (nM)	*n*	ARE_15_-G-Fl *K*_*d-app*_^b^ (nM)	*n*	Δ*G°*_*ARE1-Fl*_^*c*^ (kcal/mol)	Δ*G°*_*ARE15-G-Fl*_^c^ (kcal/mol)	ΔΔ*G°* versus ARE1-Fl (kcal/mol)
p37wt	19 ± 3	7	110 ± 5	3	−10.5	−9.5	1.0
1-239	101 ± 8	5	176 ± 9	3	−9.5	−9.2	0.3
78-287	214 ± 18	5	1094 ± 69	3	−9.1	−8.1	1.0
78-239	505 ± 34	6	796 ± 103	3	−8.6	−8.3	0.3
69-287	59 ± 9	4	467 ± 33	3	−9.9	−8.6	1.2
69-239	527 ± 54	4	933 ± 90	3	−8.6	−8.2	0.3

a Proteins described in Figure 2*A*. All include N-terminal His_6_ tags.

b Apparent equilibrium dissociation constants were resolved using fluorescence anisotropy–based binding assays by nonlinear regression to Equation 1 where *K*_*d-app*_ = 1/*K* as described in the legend for Figure 2*D* and are listed as the means ± SD of *n* independent experiments. Dissociation constants listed for the ARE1-Fl substrate are copied from Table 2 for comparative purposes.

c Free energy of protein binding to indicated RNA ligands was calculated as Δ*G*° = -*RT*ln*K*.

### The C-terminal domain of His_6_-p37^AUF1^ is required for RNA remodeling activity

**Figure 5:**
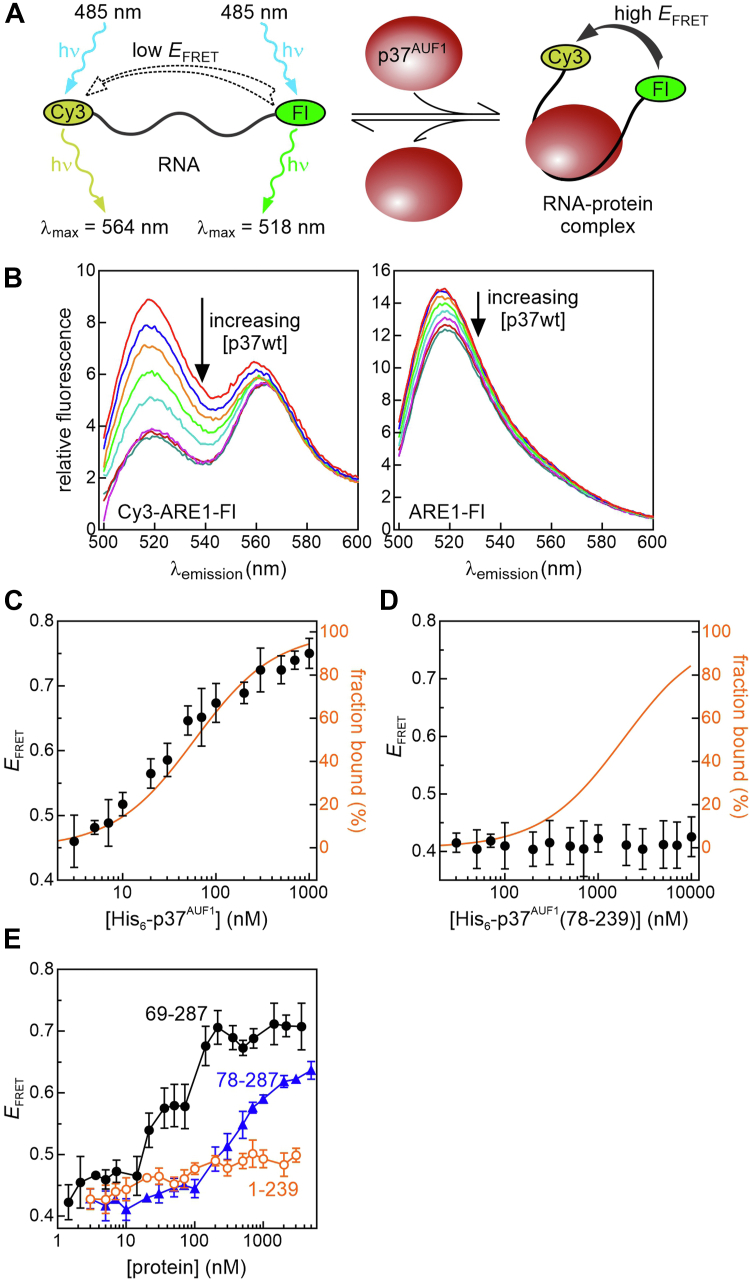
**RNA conformational remodeling by His_6_-p37^AUF1^ and select mutants resolved using FRET.***A*, schematic of the FRET assay using a dual-labeled ARE1 RNA substrate. When the Cy3-ARE1-Fl ligand is unfolded (*left*), the large distance between the fluorescein (Fl) donor and cyanine-3 (Cy3) acceptor yield low FRET efficiency (*E*_FRET_). When His_6_-p37^AUF1^ binds to this RNA substrate, it induces a condensed RNA conformation detectable by high *E*_FRET_. *B*, fluorescence emission spectra of RNA substrate ARE1 (2 nM) linked to a 3′-Fl FRET donor fluorophore in the presence (*left panel*) or absence (*right panel*) of a 5′-Cy3 acceptor dye, each incubated with various concentrations (0–1000 nM) of His_6_-p37^AUF1^. *C*–*E*, protein-dependent changes in the distance between the termini of the Cy3-ARE1-Fl RNA ligand were assessed by measuring *E*_*FRET*_ across titrations of indicated proteins using Equation 8 as described in Experimental procedures. Points represent the mean ± SD of at least three independent reactions. For His_6_-p37^AUF1^ (*C*) and His_6_-p37^AUF1^(78-239) (*D*), RNA fractions bound as a function of protein concentration were plotted using Equation 1 with binding constants calculated from anisotropy experiments performed using the same buffer system (*orange lines*).

When AUF1 binds RNA, it induces local condensation of RNA structure, which we have previously demonstrated by measuring protein-dependent changes in the distance between the termini of RNA ligands using FRET (16, 33). In particular, His_6_-p37^AUF1^ binding induced folding of the ARE1 RNA substrate but not a truncated version lacking the 5′ non-ARE sequence extension (18). This led to the hypothesis that p37^AUF1^ molecules remodel local RNA structure by binding RNA targets at multiple points, possibly using multiple protein domains. To test this theory, we measured the ability of select p37^AUF1^ mutants to fold an ARE1 RNA that was 3′-labeled with Fl (FRET donor) and 5′-labeled with cyanine-3 (Cy3; FRET acceptor) (Fig. 5*A*) by measuring FRET efficiency (*E*_FRET_) between these dyes across a titration of protein concentration. As expected, increasing concentrations of full-length His_6_-p37^AUF1^ protein significantly decreased fluorescence emission from the FRET donor but only when the acceptor was also present (Fig. 5*B*, *cf.* peak at 520 nm in left *vs.* right panels). Calculating *E*_FRET_ at each protein concentration revealed that His_6_-p37^AUF1^ induced a folded conformation on the Cy3-ARE1-Fl RNA, proportionate to the degree of ligand saturation calculated from anisotropy-derived binding constants (Fig. 5*C*, *orange line*). By contrast, the tandem RRM domain alone had no effect on folding of the Cy3-ARE1-Fl substrate (Fig. 5*D*), indicating that one or both flanking domains of p37^AUF1^ are required for its RNA remodeling activity. Including the N-terminal domain (His_6_-p37^AUF1^(1-239)) did not alter the overall conformation of the Cy3-ARE1-Fl ligand (Fig. 5*E*, *orange open circles*). However, restoring the C-terminal domain (His_6_-p37^AUF1^(78-287)) was sufficient to rescue most of the RNA remodeling activity (Fig. 5*E*, *blue triangles*). When the nine residues prior to the N-terminal RRM were also included along with the C-terminal domain (His_6_-p37^AUF1^(69-287)), RNA remodeling was restored to the same level as that seen with the full-length protein (Fig. 5*E*, *black closed circles*). These data show that the p37^AUF1^ C-terminal domain is required for altering the local conformation of bound RNA ligands, although sequences immediately upstream of the N-terminal RRM may also contribute to the final folded state.

### The microenvironment near a tryptophan residue in the p37^AUF1^ C-terminal domain is significantly altered upon binding the ARE1 RNA ligand

**Figure 6:**
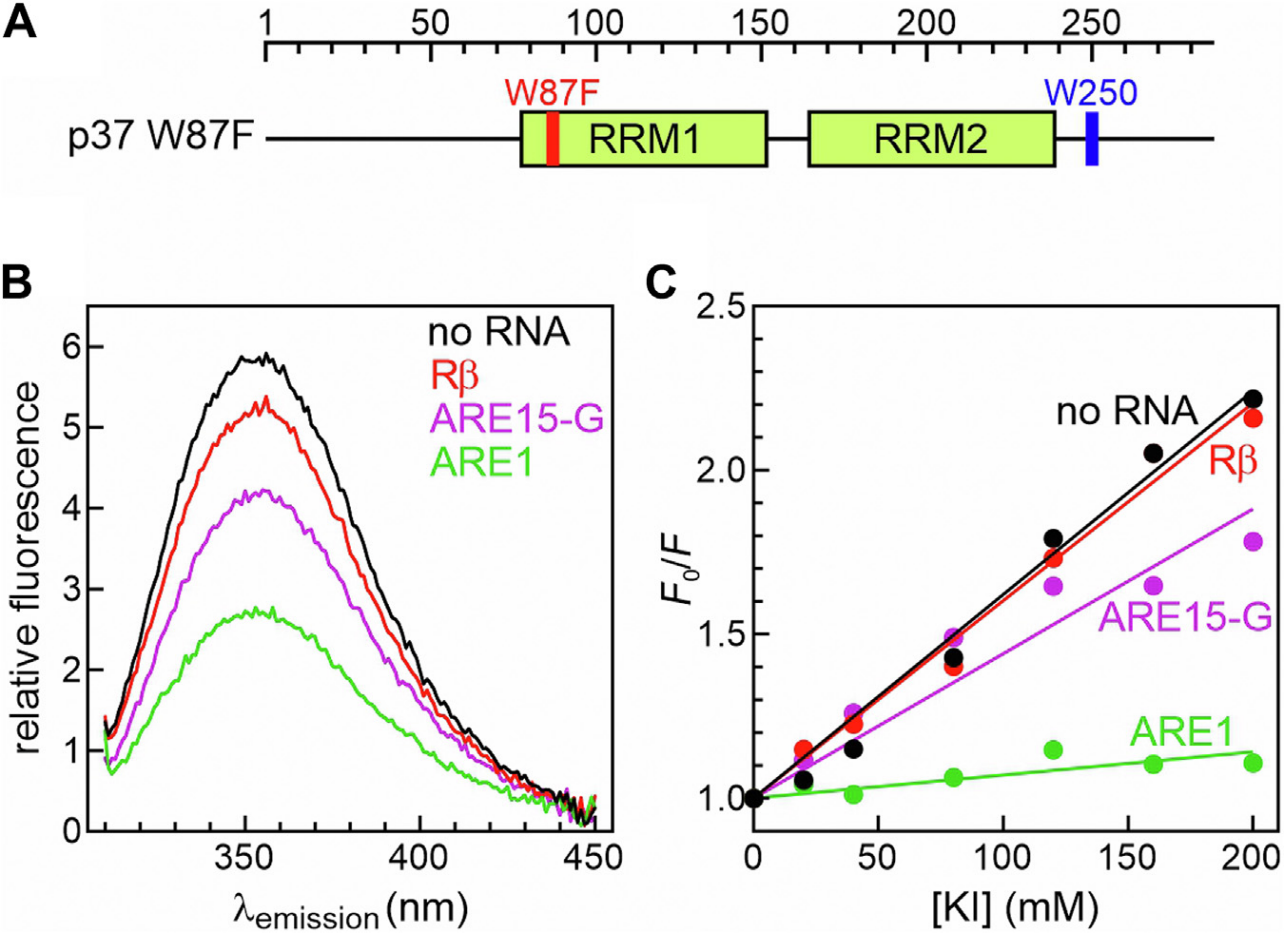
**Effects of RNA substrates on fluorescence of a Trp residue in the p37^AUF1^ C-terminal domain.***A*, schematic of His_6_-p37^AUF1^ W87F mutant showing locations of the tryptophan-to-phenylalanine substitution (*red*) and the position of the remaining tryptophan residue (*blue*). *B*, emission spectra (λ_ex_ = 295 nm) of 2 μM His_6_-p37^AUF1^ W87F alone (*black*) or in the presence of equimolar concentrations of unlabeled RNAs ARE1 (*green*), Rβ (*red*), or ARE_15_-G (*purple*). *C*, Stern-Volmer plots prepared from fluorescence emission measurements (λ_ex_ = 295 nm, λ_em_ = 350 nm) of His_6_-p37^AUF1^ W87F (2 μM) alone or in the presence of equimolar concentrations of listed RNAs after mixing with varying concentrations of potassium iodide (KI). Stern-Volmer constants were calculated using Equation 9 and are listed in Table 6.

Based on the accumulated data showing critical roles for the p37^AUF1^ C-terminal domain in binding and remodeling RNA substrates, a tryptophan residue at position 250 was utilized to interrogate RNA-dependent changes in the local chemical environment. The two tryptophan residues of p37^AUF1^ at positions 87 and 250 were previously described (Fig. 2*A*). For these experiments, a full-length protein where W87 was mutated to phenylalanine (His_6_-p37^AUF1^ W87F, Fig. 6*A*) was generated and purified (Fig. S2*A*). This protein retains the ability to bind the ARE1-Fl substrate albeit with a small energetic penalty (*K*_*d-app*_ = 50 ± 4 nM, Fig. S2*B*) but also allows the fluorescence of W250 to be exclusively monitored to detect changes in its local environment within the C-terminal domain when the protein binds to RNA. To ensure that tyrosine residues scattered through His_6_-p37^AUF1^ were not contributing significant background signal, a double mutant was created and purified (His_6_-p37^AUF1^ W87F W250F, Fig. S2*A*), and its fluorescence analyzed under conditions identical to the W87F single-site mutant protein. His_6_-p37^AUF1^ W87F W250F yielded a small, blue-shifted emission peak characteristic of tyrosine fluorescence, but the intensity was minimal compared to emission from the His_6_-p37^AUF1^ W87F protein, whose spectrum is therefore heavily dominated by fluorescence from W250 (Fig. S2*C*).

In the absence of RNA ligands, fluorescence from His_6_-p37^AUF1^ W87F yielded a characteristic tryptophan emission peak at 350 nm (Fig. 6*B, black line*). Adding an equimolar concentration of the nonbinding Rβ RNA altered emission only slightly (Fig. 6*B*, *red line*). However, titration of unlabeled ARE1 substrate led to proportional suppression of tryptophan emission, approaching saturation at a protein:RNA ratio of 1:1 (Fig. S2*D*), with minimal changes in fluorescence as RNA concentration increased further. At equimolar ARE1 concentrations, emission from His_6_-p37^AUF1^ W87F was suppressed by over 50% (Fig. 6*B*, *green line*), indicating a significant RNA ligand-dependent modulation of the microenvironment around W250. Independently, iodide quenching experiments were performed to test whether RNA ligands altered the solvent accessibility of W250. In His_6_-p37^AUF1^ W87F samples lacking or containing the nonbinding Rβ RNA, fluorescence from W250 was quenched very efficiently by iodide (Fig. 6*C*, *black and red*), denoted by the high Stern-Volmer constants (Table 6) and indicating a high degree of solvent accessibility. Conversely, in the presence of the ARE1 ligand, W250 was nearly completely shielded from the quencher (Fig. 6*C*, *green*), yielding a significantly lower Stern-Volmer constant (Table 6). Together, these data indicate that interaction with the ARE1 substrate induces a conformational change and/or involves direct RNA contacts within the His_6_-p37^AUF1^ C-terminal domain.

**Table 6:** RNA ligand effects on Stern-Volmer quench constants for W250 within His_6_-p37^AUF1^ W87F

RNA ligand	*K*_SV_ (M^−1^)^a^	*n*	*P* versus no RNA
-	6.59 ± 0.33	3	-
ARE1	0.60 ± 0.19	3	< 0.0001
Rβ	6.34 ± 0.28	3	*ns*^b^
ARE_15_-G	3.88 ± 0.48	3	0.0013

a Stern-Volmer constants were calculated from iodide quenching experiments described in the legend for Figure 6*C* using Equation 9. Each value represents the mean ± SD of *n* independent experiments.

b *ns*, not significant.

RNA-protein binding studies showed that the p37^AUF1^ C-terminal domain was required for the energetic benefits (Table 5) and RNA remodeling activity (Fig. 5*E*) of base-independent contacts 5′ of the core ARE sequence in the ARE1 ligand. A role for this RNA domain in binding and/or remodeling the p37^AUF1^ C-terminal region was orthogonally demonstrated by protein fluorescence and quenching measurements in the presence of the ARE_15_-G substrate, which lacks this 5′ extension. While His_6_-p37^AUF1^ binding to ARE_15_-G-Fl is approximately 6-fold weaker than to the ARE1-Fl substrate (Table 5), protein fluorescence experiments were performed at sufficiently high concentrations of protein and RNA (2 μM) to ensure saturation. The ARE_15_-G ligand had significantly smaller effects on fluorescence emission (Fig. 6*B*, *purple line*) and solvent accessibility (Fig. 6*C*, *purple* and Table 6) of W250 than those observed for ARE1 RNA when bound to His_6_-p37^AUF1^ W87F. These data indicate that, without the base-independent contacts 5′ of the core AU-rich binding site, the W250 microenvironment is incompletely modified, further supporting a functional interaction between these nonspecific RNA nucleotides and the His_6_-p37^AUF1^ C-terminal domain.

## Discussion

To understand the mechanism(s) by which AUF1 identifies and binds RNA substrates, it is necessary to define the contributions made by different segments of the protein. Owing to the predicted intrinsic disorder of all sequences beyond the RRM modules, there are minimal structural cues to guide identification of candidate functional protein domains (Fig. 1). This also likely explains the failures to crystallize any AUF1 protein beyond the N-terminal RRM (31), since conformational heterogeneity within the intrinsically disordered domains would impede adoption of the regular stable structures required for crystal formation. This study used quantitative biochemical approaches to identify protein domains contributing to p37^AUF1^’s RNA-binding and RNA-remodeling functions.

A previous report described roles for p37^AUF1^ flanking domains in ARE binding (28) but was hampered by misassignment of the RRM domains and reliance on EMSAs for the quantitation of highly dynamic RNA–protein equilibria. An additional challenge was that these studies used extended (>140-nt) RNA substrates that precluded dissection of initial *versus* subsequent protein-binding events (46). Nonetheless, data from the current study does affirm some conclusions from the previous reports, including the following: (i) that the tandem RRM domain alone is unable to bind RNA ligands with high affinity (Fig. 2, Table 2), (ii) that folding of the tandem RRM domain is not strengthened by flanking peptide sequences (Fig. 3, Table 3), and (iii) that both the N- and C-terminal domains contribute to stabilization of RNP complexes on ARE substrates (Table 2). However, data from the current study contradict the previous work in some areas and extend its findings in others. For example, we found that the tandem RRM domain alone bound an RNA ligand approximately 25-fold weaker than the full-length p37^AUF1^ protein (Table 2). By contrast, the previous report indicated an almost 300-fold difference in affinity between these proteins (28), but we suggest that much of this difference may result from the assignment of RRM2 ending at position 229, which would remove the entire fourth β-strand from this domain (Fig. 1*A* and Ref. (29)). Another distinction between the studies regards the roles of specific subsections of the N-terminal domain in stabilizing RNP complexes. Deleting a 28-residue alanine-rich region from p37^AUF1^ was previously reported to decrease ARE-binding affinity by almost 8-fold (28), while removing a comparable (29-amino acid) sequence had minimal impact on RNA-binding affinity in this study (Table 2). Rather, our N-terminal truncation series revealed that the nine residues immediately upstream of the N-terminal RRM contributed over half of the binding energy from the entirety of the N-terminal domain (Table 2). Intriguingly, this energetic benefit was only observed when the protein C-terminal domain was present, suggesting an allosteric interaction between these flanking sequences that enhances RNA-binding activity beyond what either of those motifs contribute independently (Table 2). Finally, the use of fluorescence anisotropy–based binding assays in this study, coupled with shorter RNA substrates with defined numbers of AUF1-binding events, also allowed the contributions of flanking protein domains to each stage of RNP formation to be quantified. Binding experiments with the Fl-ARE2 ligand showed that RNA-induced protein oligomerization (*K*_*2-app*_) was driven almost entirely by the C-terminal domain (Table 4). However, comparing the sum of flanking domain contributions to the second binding step (ΔΔ*G*^*°*^_*2*_ = 1.2 kcal/mol) with the penalty observed for the tandem RRM domain alone (>2.8 kcal/mol) suggests potential redundancy in the roles of flanking domains in the formation of p37^AUF1^ oligomers on ARE substrates.

In previous work, macromolecular binding density analysis resolved an apparent site size for the initial His_6_-p37^AUF1^ binding event on an ARE ligand as 34 ± 2 nt, which was confirmed by observations that binding was significantly weakened on shorter RNA substrates (16). In a subsequent work, the nucleotide content of a high-affinity p37^AUF1^ substrate was dissected to reveal a core 15 nt of AU-rich RNA flanked by a 3′-purine and an extended (19 nt) 5′-tract that stabilized protein:RNA complexes in a largely sequence-independent manner (18). Furthermore, RNP stabilization by this 5′ extension included a significant negative change in heat capacity commonly associated with induced-fit binding mechanisms between proteins and nucleic acid substrates (47, 48), but not with formation of new ionic contacts, excluding protein interactions with the phosphodiester backbone as likely contributing factors. Finally, contacts with the 5′ ligand segment were required for p37^AUF1^-directed condensation of local RNA structure (18). Based on these observations, we hypothesized that one or both of the intrinsically disordered flanking domains of p37^AUF1^ may stabilize RNP complexes by making small, base-independent interactions with this stretch of RNA. Three major observations from this study support roles for the p37^AUF1^ C-terminal domain in mediating the RNP-stabilizing and RNA-remodeling activities of this 5′ RNA sequence. First, when the 5′ non-ARE extension was removed from the ARE1 ligand (ARE_15_-G, Table 1), only p37^AUF1^ mutants that contained the C-terminal domain suffered a severe penalty in binding affinity (Table 5), implicating this domain as the primary partner in RNP stabilization *via* the 5′ tract. Second, this model is supported by our fluorescence studies with the p37^AUF1^ W87F mutant protein, which evaluated the microenvironment near W250, positioned within the C-terminal domain. When bound to an RNA ligand containing the 5′-extended sequence (ARE1), fluorescence from W250 was severely quenched and was sequestered from solvent (Fig. 6, Table 6). However, RNA-dependent repression of both fluorescence quantum yield and solvent accessibility were significantly attenuated when bound to an RNA ligand lacking the 5′ extension. Finally, FRET-based RNA-folding experiments demonstrated that p37^AUF1^ mutants lacking the C-terminal domain were incapable of condensing the conformation of an RNA substrate (Fig. 5), which also requires these upstream flanking nucleotides (18). Based on these observations, we find it probable that the disordered C-terminal domain stabilizes AUF1 RNPs and remodels local RNA structure by making direct base-independent contacts with RNA sequences 5′ of a nucleating AU-rich tract. However, our data do not completely exclude the possibility that these events are linked by an indirect allosteric mechanism.

AUF1 binding to RNA targets is associated with several functional outcomes, including mRNA destabilization, mRNA stabilization, and enhanced translational efficiency (20). However, how AUF1 facilitates these outcomes remains largely unknown. One model suggests that AUF1 nucleates assembly of a multisubunit *trans*-acting complex that includes factors associated with mRNA decay (polyA-binding protein) and translation (eukaryotic translation initiation factor 4G), which in turn may ultimately recruit or restrict access to the mRNA catabolic or translation initiation machinery (19). If the disordered flanking domains of p37^AUF1^ adopted stable or quasi-stable local structures upon binding to RNA ligands as suggested by data from the W250 fluorescence experiments (Fig. 6, Table 6), these remodeled sequences could serve as docking sites for ancillary factors that ultimately execute the regulatory consequences of AUF1 recruitment. A second, nonexclusive model is that AUF1 may serve as an RNA chaperone by altering RNA conformations near its binding site to enhance or inhibit access to proximal *trans*-factor binding events (27). This model is supported by observations that AUF1 binding to c-*myc* mRNA can enhance its translation by blocking access for the translation inhibitory protein TIAR (49) but can also positively or negatively affect miRNA:RISC complex recruitment to nearby target sites (50, 51). Separate RNA contacts made by disparate domains of p37^AUF1^ may logically mediate these modifications to local RNA structure and may also explain the abnormally long RNA site size occupied by this protein (16).

While this study identifies unique roles for the p37^AUF1^ flanking domains in binding and folding RNA substrates, it also presents several limitations that will require future studies to resolve. First, while the energetic benefit of sequences 5′ of the core ARE in the ARE1 ligand clearly required the p37^AUF1^ C-terminal domain (Table 5), it is conceivable that some RNA sequence preferences or biases exist for this interaction, which could contribute to the RNA context-dependence of AUF1 function. Second, our fluorescence data suggest that at least some subsections of the AUF1 C-terminal domain may adopt structure during RNA binding (Fig. 6, Table 6), but rigorous structural analyses (*e.g.*, by NMR) will be required to affirm this finding and to establish the degree of conformational restriction imposed during RNP assembly. To date, we have no information on potential conformational changes involving the N-terminal domain. Finally, this study focused exclusively on the p37 isoform of AUF1, but the peptide inserts that distinguish the larger isoforms all occur within the protein flanking domains. As such, it is likely that modulation of RNA contacts involving these sequences is the biochemical basis for isoform-specific differences in their RNA-binding and RNA-remodeling activities (16). These isoform-specific contacts are likely to be of significant scientific interest; for example, RNA remodeling by p45^AUF1^ is important for the replication of some flaviviruses and requires an RGG/RG-rich motif located within its C-terminal flanking domain (52, 53). It is also of note that protein sequence encoded by exon 2 (present in p40^AUF1^ and p45^AUF1^) is inserted directly upstream of p37^AUF1^ glycine 78 (13), displacing p37^AUF1^ residues 69-77, which contribute 0.8 to 0.9 kcal/mol to ARE1-binding activity when the C-terminal domain is present (Table 2), by 19 amino acids. Accordingly, the positions of sequences inserted by exons 2 and 7 in the larger AUF1 isoforms have the potential to modify not only direct contributions of the N- and C-terminal flanking domains to RNP assembly but also allosteric interactions that likely occur between these domains.

## Experimental procedures

### RNA substrates

RNA substrates used in this study were synthesized by MilliporeSigma. Sequences are listed in Table 1. Labeled variants included terminal biotin (B), Fl, and cyanine-3 (Cy3) groups. Dried pellets were dissolved in 10 mM Tris-HCl (pH 8.0) and quantified by A_260_ using extinction coefficients provided by the company and Beer-Lambert’s law. For Fl and/or Cy3-labeled RNA substrates, contributions of the dyes to A_260_ and the fractional concentrations of each label were calculated from measurements of absorbance at 493 nm (for Fl) and 552 nm (for Cy3) as described previously (33).

### Preparation of recombinant proteins

Complementary DNA fragments encoding truncation mutants of human p37^AUF1^ were generated *via* PCR from plasmid pBAD/HisB-p37^AUF1^ (54), incorporating unique restriction sites (Acc65I, HindIII) at fragment termini. The coding sequence of the Δ69-77 internal site mutant was synthesized by Genscript with similar terminal restriction sites. PCR products and the Δ69-77 mutant plasmid were digested and released coding sequence fragments subcloned into a pBAD/His vector (Thermo Fisher Scientific) that had been modified to include the truncated N-terminal tag MSHHHHHHGT. Plasmids encoding the W87F, W250F, and W87F W250F mutants of p37^AUF1^ were generated using oligonucleotide-directed PCR mutagenesis (55). Fidelity of all subcloned inserts was verified by restriction digests and Sanger sequencing.

*Escherichia coli* BL21 Rosetta 2 (DE3) cells (MilliporeSigma) transformed with pBAD plasmids expressing His_6_-tagged p37^AUF1^ wt, 78-287, 30-287, 69-287, Δ69-77, W87F, W250F, and the W87F W250F double mutant were grown in SOB media and induced using arabinose (0.02%). Proteins expressed from pBAD plasmids encoding His_6_-p37^AUF1^ mutants 78-239, 69-239, and 1-239 were similarly induced but using *E. coli* TOP10 cells (Thermo Fisher Scientific). Postinduction cultures were lysed and His_6_-tagged proteins purified by Ni^2+^ affinity chromatography.

Data from some early studies suggested that AUF1 proteins formed dimers in solution (17, 28); however, more recent work with highly purified proteins indicate that they are likely monomers (50). We hypothesize that the repeated freeze-thaw cycles used to lyse bacteria in the early studies (56) may have resulted in the formation of soluble aggregates that were retained during the purification process. Accordingly, we have modified our lysis and purification protocols to minimize aggregate formation and maximize protein purity. For native protein purifications, bacterial pellets were resuspended in buffer 1 (50 mM sodium phosphate, 500 mM NaCl, 20 mM imidazole, pH 8.0) containing 5% PEG 6000, which has been reported to increase protein solubility and inhibit aggregation in other systems (57, 58), and a protease inhibitor cocktail (10 μg/ml leupeptin, 10 μg/ml pepstatin A, and 0.1 mM PMSF). Cells were lysed by sonication on ice and debris cleared by centrifugation (20,000*g*, 40 min, 4 °C). A HiTrap IMAC column (GE Healthcare) was washed with eight column volumes of deionized water and charged with six volumes of 20 mM NiCl_2_. It was then rinsed with a further eight volumes of deionized water and six volumes of native imidazole elution buffer (10 mM sodium phosphate, 0.5 M NaCl, 0.5 M imidazole, pH 6.3) before equilibration with eight volumes of buffer 1. The clarified lysate was then loaded onto the column, which was then washed with buffer 1 until A_280_ returned to baseline. The column was then washed with six volumes of buffer 1 containing 1% Triton X-100, then again with buffer 1 alone until A_280_ returned to baseline before a pre-elution step of six volumes of 0.2 M imidazole wash buffer (10 mM sodium phosphate, 0.5 M NaCl, 0.2 M imidazole, pH 6.3) was applied. Pilot experiments revealed that both the Triton and 200 mM imidazole washes removed weakly bound contaminants, improving protein purity (*data not shown*). His_6_-tagged AUF1 proteins were then eluted using native imidazole elution buffer.

For CD experiments, His_6_-p37^AUF1^(78-239) was purified under denaturing conditions to remove a trace copurifying protein, then refolded on-column using a reverse urea gradient (6–0.2 M) as described (59). Anisotropy-based binding assays showed no significant difference in affinity between His_6_-p37^AUF1^(78-239) purified under native versus denaturing conditions for the ARE1-Fl RNA ligand (*data not shown*). All purified proteins were then desalted using a HiPrep 26/10 desalting column (GE Healthcare) into 10 mM Hepes-KOH (pH 7.5) containing 150 mM KCl and concentrated using Amicon Ultra (MilliporeSigma) or Pierce (Thermo Fisher Scientific) centrifugal filter units with either 10 kDa or 30 kDa cutoffs depending on protein molecular weight. Protein purity was assessed *via* SDS-PAGE stained with Coomassie Blue (*e.g.*, Fig. 2*B*) and yields quantified *via* absorbance at 280 nm. Extinction coefficients (Table S1) were determined using ProtParam software (60) and concentrations calculated using the Beer-Lambert law, assuming that proteins were monomeric. Dynamic light scattering (Zetasizer Nano series) analyses verified the absence of aggregates, revealing particles of 6 to 8.5 nm in diameter (depending on the protein) that comprised >99% of the total volume (Fig. S3).

Where indicated, the His_6_ tag was removed from His_6_-p37^AUF1^ using the Novagen Enterokinase Cleavage Capture Kit (MilliporeSigma). Briefly, 80 μg of His_6_-p37^AUF1^ was incubated with 4 units of EK in 1 × cleavage/capture buffer provided in the kit for 4 hours at room temperature. EK was then removed by incubation with EKapture agarose beads and captured using a spin filter. Recovered p37^AUF1^ was then quantified *via* absorbance at 280 nm and analyzed by SDS-PAGE gel to validate completion of the cleavage reaction and to assess protein purity (Fig. S1*A*).

### Electrophoretic mobility shift assays

Biotin-tagged RNA substrates (1 nM) were incubated with titrations of indicated proteins in 10 mM Tris–HCl (pH 8.0) including 100 mM KCl, 0.5 mM EDTA, 0.1 μg/μl acetylated BSA, 1 μg/μl heparin, 2 mM DTT, and 10% glycerol (20 μl final volume) on ice for 15 min. RNA–protein complexes were then crosslinked on ice under UV light (254 nm) using a Spectrolinker XL-1500 UV crosslinker (Spectronics) to 100 mJ/cm^2^. Samples were then fractionated through 8% native polyacrylamide gels at 4 °C in 0.5 × TBE (1 × TBE is 90 mM Tris-borate (pH 8.3) containing 2 mM EDTA). Gel contents were then transferred to a positively charged nylon membrane by capillary flow in 20 × SSC (1 × SSC is 15 mM sodium citrate (pH 7.0) containing 150 mM NaCl) and crosslinked at 254 nm to 120 mJ/cm^2^. Positions of biotin-tagged RNA ligands were then detected using the Thermo Chemiluminescent Nucleic Acid Detection kit (Thermo Fisher Scientific) following the manufacturer’s instructions and visualized using an Azure 300Q imaging system (Azure Biosystems).

### Fluorescence anisotropy–based binding experiments

Quantitative measurements of RNA–protein interactions were performed using an equilibrium-based fluorescence anisotropy assay essentially as described previously (17, 61). Briefly, RNA substrates (0.2–0.4 nM) labeled with Fl on either the 5′- or 3′-end were incubated with various concentrations of indicated proteins in the same buffer used for EMSA experiments (above) but lacking glycerol and in a total volume of 100 μl. KCl concentration was decreased to 50 mM in reactions containing the Fl-ARE2 substrate to minimize the cation-stabilized folding previously demonstrated for this RNA ligand (54, 62). Samples were incubated for 1 min at 25 °C before reading fluorescence anisotropy of Fl-tagged RNA substrates using a Beacon 2000 fluorescence polarization system (Panvera) equipped with fluorescein excitation (490 nm) and emission (535 nm) filters. Pilot experiments demonstrated that p37^AUF1^ binding to RNA substrates attains equilibrium within this time ((16) and *data not shown*).

Because the fluorescence quantum yield of Fl-labeled RNA substrates was not significantly modulated by any of the proteins tested (*data not shown*), apparent binding affinities were calculated from anisotropy isotherms of total measured anisotropy (*A*_*t*_) as a function of protein concentration ([P]) by nonlinear regression with PRISM software (GraphPad) using Equation 1 or 2 (17, 61).

(Eq. 1) $$A_{t}=\frac{A_{R}+A_{\mathrm{PR}}K\left[P\right]}{1+K\left[P\right]}$$

(Eq. 2) $$A_{t}=\frac{A_{R}+A_{\mathrm{PR}}K_{1}\left[P\right]+A_{P_{2}R}K_{1}K_{2}{\left[P\right]}^{2}}{1+K_{1}\left[P\right]+K_{1}K_{2}{\left[P\right]}^{2}}$$

Equation 1 describes a single-site binding model where *A*_R_ is the intrinsic anisotropy of the unbound Fl-labeled RNA, *A*_PR_ is the intrinsic anisotropy of the single protein:RNA complex, and *K* is the equilibrium association constant. The apparent dissociation constant *K*_*d-app*_ is then solved as 1/*K*. However, where two sequential binding events are indicated, Equation 2 can solve the equilibrium association constants for both binding steps (*K*_1_, *K*_2_) but also requires solution of the intrinsic anisotropy of the Fl-RNA in the saturated P_2_R complex (*A*_P2R_). To check for potential cooperativity between binding events, we used a variant of the Hill model in Equation 3, where *A*_*t*_ varies with [P] as a function of the half-maximal binding ([P]_1/2_) and the Hill coefficient (*h*) (39).

(Eq. 3) $$A_{t}=A_{R}+\left(A_{\mathrm{PR}}-A_{R}\right)\times \left[\frac{{\left(\left[P\right]/{\left[P\right]}_{1/2}\right)}^{h}}{{1+\left(\left[P\right]/{\left[P\right]}_{1/2}\right)}^{h}}\right]$$

### CD

For CD assays, recombinant His_6_-p37^AUF1^(78-239) was diluted to a final concentration of 25 μM in 10 mM sodium phosphate (pH 8.0) containing 100 mM KCl. Due to its poorer solubility, purified His_6_-p37^AUF1^ was desalted directly into this buffer, then diluted as needed to a final concentration of 25 μM. The samples were analyzed at room temperature in a Jasco J-1500 spectropolarimeter over a range of 200 to 250 nm. The lower wavelength was limited by the high-tension voltage observed at wavelengths below 200 nm. Reference spectra were recorded using buffer samples lacking protein. Protein CD spectra were obtained after subtracting reference spectra and recorded in mdeg units, which were then converted to mean residue ellipticity to normalize for concentration, residue number, and path length, using Equation 4. The units are reported in deg·cm^2^·dmol^−1^.

(Eq. 4) $${\left[\theta \right]}_{MRW,\lambda }=\frac{MRW\times \theta }{10\times d\times c}$$

Briefly, mean residue weight (MRW) equals the molecular mass divided by *N*-1, where *N* is the number of amino acids in the chain. θ is the measured ellipticity in degrees, *d* is the pathlength in cm, and *c* is the concentration in g/ml (63).

### Protein denaturation assays

To measure folded stability of select recombinant proteins, 8 μM of each was incubated in 10 mM Tris–HCl (pH 8) containing 50 mM KCl, 2 mM DTT, 0.5 mM EDTA and a range (0.2–4 M) of GnHCl concentrations. Reactions were assembled on a 60 μl scale and incubated for 1 h at room temperature. Samples were then transferred into quartz microcuvettes and protein fluorescence spectra measured following excitation at 280 nm (5 nm bandpass) in a Cary Eclipse spectrofluorometer (Varian) with emission bandpass filters set to 10 nm. To calculate the extrapolated free energy of denaturation in the absence of the denaturant (Δ*G*_*uw*_) and the sensitivity of protein-folding free energy to the denaturant (*m*_*eq*_), plots of emission at 350 nm (*F*_350_) *versus* GnHCl concentration were resolved using the linear extrapolation method as modified by Manyusa and Whitford (64) described by Equations 5 and 6 using PRISM software (GraphPad):

(Eq. 5) $$F_{350}=F_{native}-\left[\frac{\left(F_{native}-F_{unfolded}\right)\cdot e^{-\frac{\Delta G_{u}}{RT}}}{1+e^{-\frac{\Delta G_{u}}{RT}}}\right]$$

(Eq. 6) $$\Delta G_{u}=\Delta G_{uw}-m_{eq}\;\left[\text{GnHCl}\right]$$

Here, *F*_*native*_ is the fluorescence emission in the absence of denaturant, *F*_*unfolded*_ is the fluorescence emission of the fully unfolded protein, Δ*G*_*u*_ is the free energy of denaturation, *R* is the gas constant (1.987 × 10^−3^ kcal mol^−1^ K^−1^), and *T* the absolute temperature.

### Fluorescence resonance energy transfer

RNA conformational changes mediated by protein binding were assessed by measuring the distance between the 3′-Fl (FRET donor) and 5′-Cy3 (FRET acceptor) dyes on the dual-labeled Cy3-ARE1-Fl substrate using FRET, essentially as described previously (18). FRET efficiency (*E*_*FRET*_) varies with the distance between donor and acceptor fluorophores *via* Equation 7, where *r* is the scalar distance between the dyes and *R*_*0*_ is the Förster distance, or the distance at which *E*_*FRET*_ = 0.5 (65). For the Fl-Cy3 dye pair linked to single-stranded nucleic acids, *R*_*0*_ was previously calculated as 55.7 Å (66).

(Eq. 7) $$E_{\text{FRET}}=R_{0}^{6}/\left(R_{0}^{6}+r^{6}\right)$$

RNA–protein reactions were assembled in the same buffer used for anisotropy reactions with the ARE1-Fl ligand (*above*) except that KCl was lowered to 50 mM to minimize RNA folding in the absence of protein. At each protein concentration, samples included no RNA (blank), 2 nM ARE1-Fl (donor alone), or 2 nM Cy3-ARE1-Fl (donor-acceptor pair) and were incubated for 1 min at room temperature. Fluorescence was measured in a Cary Eclipse spectrofluorometer (Varian) with excitation at 485 nm (10 nm bandpass); emission was measured spectrally (500–650 nm) or at peak donor emission (λ_em_ = 520 nm) using a 10 nm bandpass. Subtracting blank emission from ARE1-Fl signals at 520 nm yielded fluorescence of the donor in the absence of the acceptor (*F*_D_), while blank subtraction from Cy3-ARE1-Fl emission at the same wavelength yielded the fluorescence of the donor in the presence of the acceptor (*F*_DA_). From these values, *E*_*FRET*_ was calculated using Equation 8, where *f* is the labeling efficiency of the FRET acceptor (Cy3) on the double-labeled RNA substrate (18).

(Eq. 8) $$E_{\text{FRET}}=1-\left[\frac{F_{\text{DA}}-F_{D}\left(1-f\right)}{F_{D}f}\right]$$

### Intrinsic protein fluorescence

Purified recombinant proteins were diluted to 2 μM in 10 mM Tris–HCl (pH 8) containing 100 mM KCl, 1 μg/μl heparin, 2 mM DTT, and 0.5 mM EDTA. Protein fluorescence was measured using a Cary Eclipse spectrofluorometer (Varian) following excitation at 295 nm (5 nm bandpass) to maximize selectivity for tryptophan (67). Emission was read spectrally (310–450 nm) or at peak emission (λ_em_ = 350 nm) using a 10 nm bandpass after subtracting signals from samples lacking protein. Where indicated, RNA ligands were added at select molar ratios. Samples were mixed for a minimum of 2 min before taking fluorescence measurements. Fluorescence quenching experiments were performed under the same conditions as above, but including various concentrations of potassium iodide (KI). The sensitivity of tryptophan fluorescence to KI was calculated by linear regression using the Stern-Volmer equation (Eq. 9), where *F*_*0*_ is peak emission in the absence of the quencher, *F*_*i*_ is emission at each quencher concentration ([Q]), and *K*_*SV*_ is the Stern-Volmer quench constant (68).

(Eq. 9) $$\frac{F_{0}}{F_{i}}=1+K_{SV}\left[Q\right]$$

### Statistics

Comparisons of affinity and quenching constants were performed using the unpaired *t* test based on the mean ± SD of each parameter from at least three independent replicate experiments. Differences yielding *p* < 0.05 were considered significant.

## Data availability

All data supporting the findings of this study are available in the article and supporting information. Additional data are available upon request from the corresponding author.

## Supporting information

This article contains supporting information (60).

## Conflicts of interests

The authors declare that they have no conflicts of interests to with the contents of this article.

## Supporting information

SI fig revised legends
