# Supplementary material for: Unstructured protein domains stabilize RNA binding and mediate RNA folding by AUF1
Source: J Biol Chem. 2025 Mar 25;301(5):108442. doi: 10.1016/j.jbc.2025.108442 (PMC12147176; doi:10.1016/j.jbc.2025.108442)
Supplement: SI fig revised legends [file mmc1.docx]

**Figure S1 The His_6_ tag does not impact p37^AUF1^ RNA-binding activity.** *A*, Coomassie blue-stained SDS-PAGE of purified His_6_-p37^AUF1^ and enterokinase (EK)-cleaved p37^AUF1^. Molecular weight markers (in kDa) are listed (*left*). *B*, Fluorescence anisotropy-based assays of His_6_-p37^AUF1^ (*black solid circles*) and EK-cleaved p37^AUF1^ (*red open circles*) binding to the ARE1-Fl RNA substrate analyzed using the single site binding model of Eq. 1.

**Figure S2 Purification and characterization of select tryptophan substitution mutants of His_6_-p37^AUF1^.** *A*, Coomassie blue-stained SDS-PAGE of purified His_6_-p37^AUF1^ W250F, His_6_-p37^AUF1^ W87F, and His_6_-p37^AUF1^ W87F W250F proteins. *B*, Representative fluorescence anisotropy assays of His_6_-p37^AUF1^ (*black, closed circles*), His_6_-p37^AUF1^ W250F (*red, open circles*), and His_6_-p37^AUF1^ W87F (*blue triangles*) binding to the ARE1-Fl RNA substrate analyzed using the single site binding model of Eq. 1. *C*, Emission spectra (λ_ex_ = 295 nm) of 2 µM His_6_-p37^AUF1^ W87F versus His_6_-p37^AUF1^ W87F W250F showing that non-tryptophan residues make minimal contributions to fluorescence. *D*, Emission spectra (λ_ex_ = 295 nm) of 2 µM His_6_-p37^AUF1^ W87F in the presence of varying molar ratios of unlabeled RNA ligand ARE1. The spectrum taken at the 1:1 protein:RNA ratio is also shown in Fig. 6*B* for reference.

**Figure S3 Size distribution of purified His_6_-p37^AUF1^ analyzed by dynamic light scattering (DLS).** A representative volume trace showing purified His_6_-p37^AUF1^ as a monodisperse species in solution with an average diameter of 8.1 nm.
