# Supplementary material for: Unstructured protein domains stabilize RNA binding and mediate RNA folding by AUF1
Source: J Biol Chem. 2025 Mar 25;301(5):108442. doi: 10.1016/j.jbc.2025.108442 (PMC12147176; doi:10.1016/j.jbc.2025.108442)
Supplement: JBC-D-24-03433.zip [file mmc2.zip › Table S1.docx]

**Table S1: Molecular weights and extinction coefficients of proteins used in this study**

| **Protein*^a^*** | **Molecular Weight (Da)** | **Extinction Coefficient (M^-1^·cm^-1^)*^b^*** |
| --- | --- | --- |
|  |  |  |
| His_6_-p37wt | 34903 | 22920 |
| EK-cleaved p37wt | 31602 | 21430 |
| 78-239*^b^* | 19710 | 9970 |
| p37 W250F | 31850 | 15930 |
| 1-239 | 26514 | 9970 |
| 78-287 | 25068 | 21430 |
| 30-287 | 29310 | 21430 |
| 69-287 | 26086 | 21430 |
| 69-239 | 20728 | 9970 |
| p37Δ(69-77) | 30871 | 21430 |
| p37 W87F | 31850 | 15930 |
| p37 W87F W250F | 31811 | 10430 |

*^a^*Mutant proteins without listed tags were appended with a truncated His_6_ tag (MSHHHHHHGT) for purification purposes and the truncated His_6_ tag was included in the molecular weight. Contributions of the truncated His_6_ tag to calculated extinction coefficients were negligible.

*^b^*Extinction coefficients at 280 nm were calculated using the ProtParam tool (57), assuming that all Cys residues were reduced.
