# Supplementary figures and images for: Unstructured protein domains stabilize RNA binding and mediate RNA folding by AUF1
Source: J Biol Chem. 2025 Mar 25;301(5):108442. doi: 10.1016/j.jbc.2025.108442 (PMC12147176; doi:10.1016/j.jbc.2025.108442)

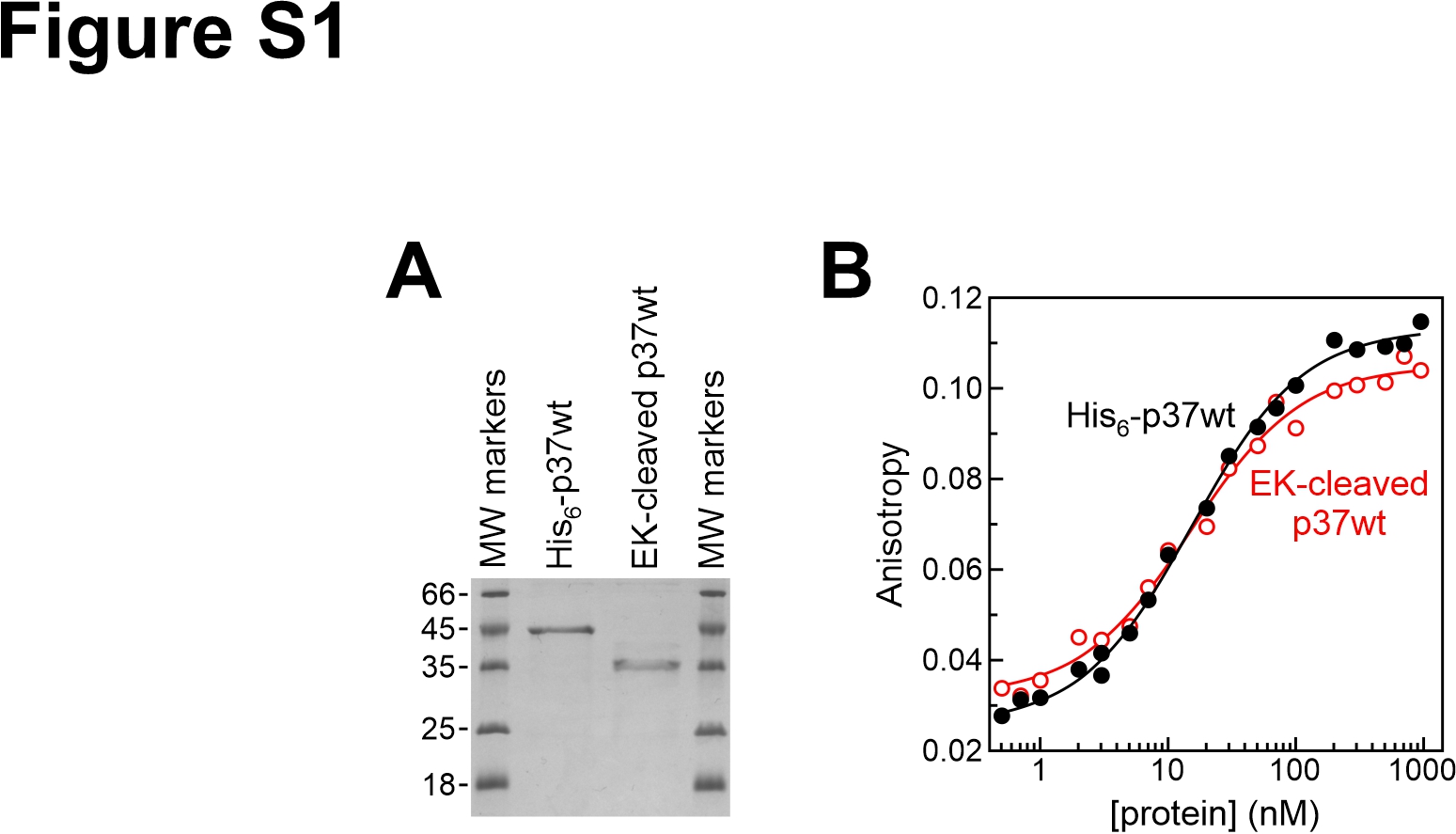

Supplement: JBC-D-24-03433.zip [file mmc2.zip › Fig S1.jpg]

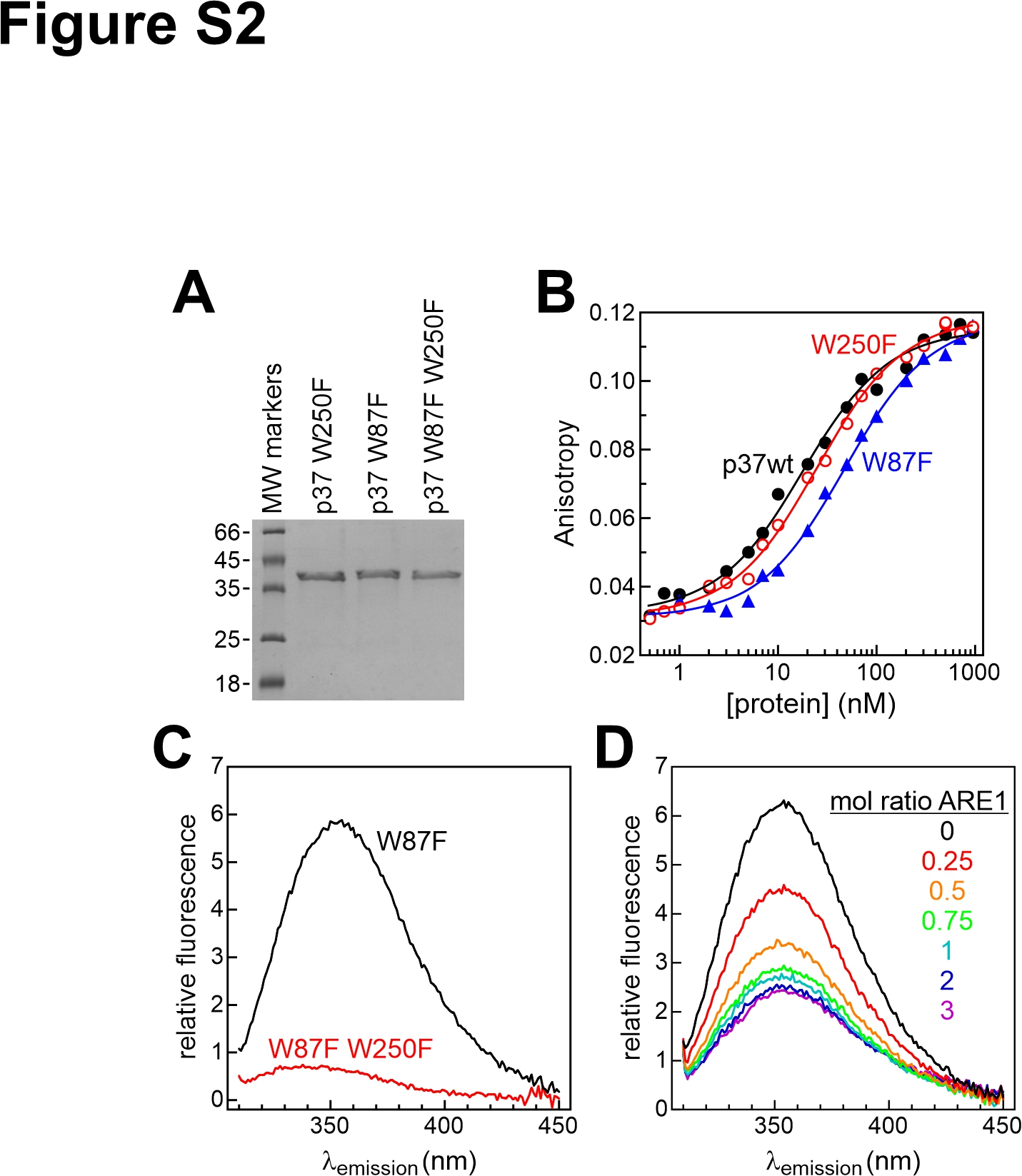

Supplement: JBC-D-24-03433.zip [file mmc2.zip › Fig S2.jpg]

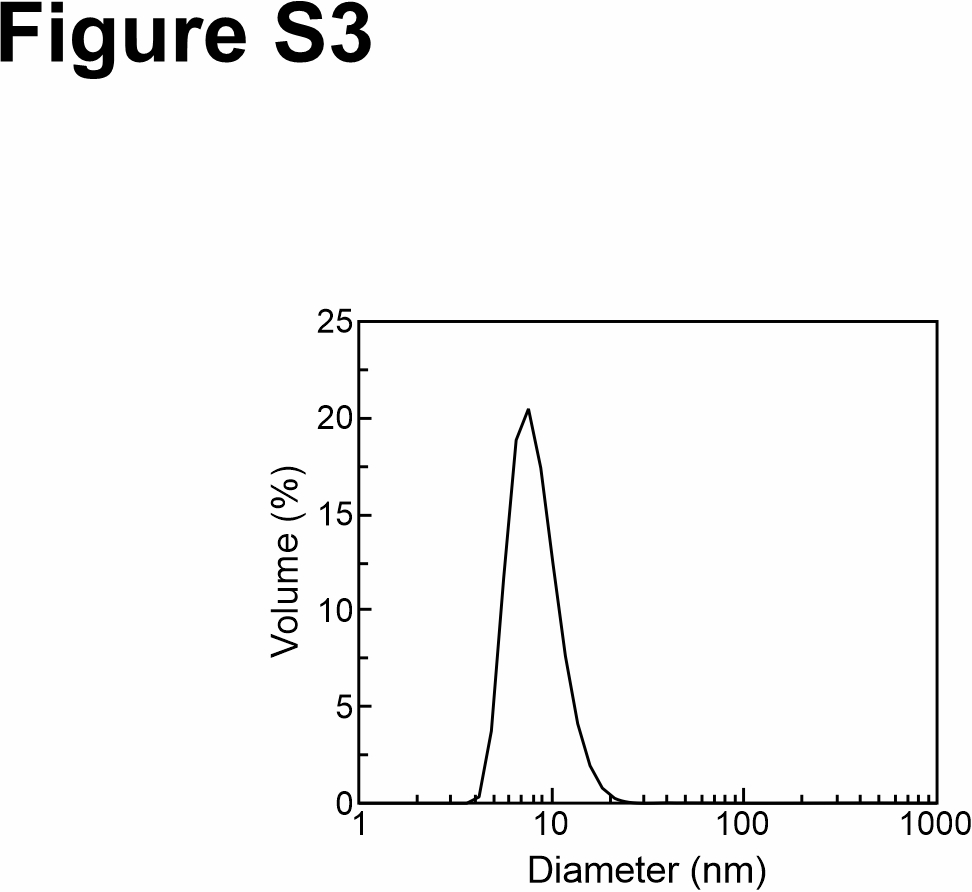

Supplement: JBC-D-24-03433.zip [file mmc2.zip › Fig S3.jpg]
